# Factors associated with needlestick injuries among healthcare workers: implications for prevention

**DOI:** 10.1186/s12913-021-07110-y

**Published:** 2021-10-09

**Authors:** Kifah Habib Alfulayw, Sultan T. Al-Otaibi, Hatem A. Alqahtani

**Affiliations:** 1 Department of Family and Community Medicine, College of Medicine, Imam Abdulrahman Bin Faisal University, Dammam, Saudi Arabia; 2 Department of Public Health, College of Public Health, Imam Abdulrahman Bin Faisal University, PO Box 1982, Dammam, 31441 Saudi Arabia

**Keywords:** Needle stick injury, Training, Health-care workers

## Abstract

**Background:**

Our study sought to determine the frequency of Needlestick injuries (NSIs) among Healthcare Workers (HCWs) working at governmental hospital and to study the factors that associated with occurrence of NSIs, and to develop recommendations for a comprehensive program for prevention.

**Methods:**

Retrospective study of all reported cases of NSIs in the period from April 2016 to May 2018 among healthcare workers at a governmental hospital.

**Results:**

Incidence of NSIs over 26 months was 8.4% among all participants. Nurses were the most affected staff (52.5%) resulted commonly from disposing syringes (58.9%). In contrast, the incidence of NSIs among physicians was 24.9% where surgical devices were the primary source of NSIs among them (40%). Failure to complete all required hepatitis B vaccination was common among expatriates of the participants of this study.

**Conclusions:**

NSIs was common among HCWs participated in this study. Preventive measures should be implemented including adequate hepatitis B immunization.

## Introduction

Needlestick injuries (NSIs) exposing workers to blood borne pathogens pose a major risk to healthcare workers. These incidents can transmit many blood-born infectious diseases, especially viruses. These include hepatitis B (HBV), hepatitis C (HCV) and human immunodeficiency virus (HIV). Despite implementation of preventive measures to reduce sharp injuries (such as equipment design improvement and employee training), they continue to occur in every step of sharp devices usage, disassembly, or disposal. The US Occupational Health and Safety Administration (OSHA) estimated that 5.6 million of healthcare workers (HCWs) are at risk of occupational exposure to different blood-borne pathogens due to NSIs [1].

Sharp injury can occur whenever a worker is exposed to contact with sharp object, that may result in harm. Sharps and needlestick injuries are wounds caused by needles and other sharp medical instruments (e.g., scalpel, blades, and scissors) that accidentally puncture or cut the skin. Sharps and needles may only cause small wounds in the skin, but the effects can be worse. Such instruments come in contact with blood and other body fluids and may carry the risk of infections. If contaminated device punctures skin of healthcare workers (HCWs), they face a high risk of occupational exposure to infected, hazardous fluids.

The burden of needlestick and sharp injuries is worldwide. A study done in 52 ministry of health hospitals during 2012 in the Kingdom of Saudi Arabia estimated that the annual sharp injury rate was 3.2 per 100 occupied beds. Nurses were the job category most affected by NSIs, wards were the most common location of NSIs, disposable syringes were the primary source caused injury, and most of injuries occurred during device use [2]. Memish also reported that in United States, the rate of sharp injuries per 100 occupied beds is 20.7 for teaching hospitals and 16.5 for non-teaching hospitals [3]. In addition, according to Center of Disease Control and Prevention (CDC), 385,000 sharp injuries occur annually in the US among hospital workers [1]. On a global level, according to World Health Organization (WHO), 3 million healthcare workers are exposed annually to percutaneous fluid contaminated with at least hepatitis B (about 2,000,000 exposures), HIV (approximately 170,000 exposures), and hepatitis C (about 900,000 exposures) [4].

The Ontario Hospital Association/Ontario Medical Association estimated that after a needlestick injury from a needle contaminated with HBV, there is a 6–30% chance that an exposed susceptible person will be infected. In a similar situation with hepatitis C, the chance of infection of the exposed worker is 1.8%, and the chance with HIV is 0.3% [5]. After exposure to infected blood following NSIs, infection risk to the employee depends on involved blood-borne pathogen, sharp injury severity, employee immune status, and usage of suitable and correct prophylaxis after injury [6].

Several studies considered determinants of NSIs. Many factors increase risks of NSIs, such as recapping needles, overuse and unnecessary use of sharp devices, lack of device with safety measures, absent of personal protective equipment and containers for sharp disposal, lack of engineering control (e.g. needles with safety features), shortage of staff, lack of training, inappropriate disposal of sharp device, and patient reactions [7]. In addition, factors contributing to NSIs include inadequacy of protective and safety measures in medical devices at work [8].

A study done among nurses showed that there is a significant association between the quantity of needles used each day and the probability of needlestick injury. In addition, extended schedules of work increase the risk of needlestick injury. Needlestick injuries were significantly associated with working hours, particularly if employee work at least once per week for 13 h or more [9].

It is critically important to assess determinants of sharp injuries to guide implementing effective prevention standards and programs. Our study sought to determine the frequency of NSIs among HCWs working in Dammam Medical Complex, to study the factors that associated with occurrence of NSIs, and to develop recommendations for a comprehensive program for prevention.

## Methods

A retrospective analysis of all reported cases of needlestick injuries in the period from April 2016 to May 2018 in the Dammam Medical Complex in Dammam City, Eastern Province, Saudi Arabia was conducted. Information was derived from the EPINet (Exposure Prevention Information Network) software, which provides standardized fields to record, monitor and analyze needlestick injuries and body fluids contacts. This software records comprehensive and detailed information about each sharp device incident. EPINet reports include job category, nationality, time of injury, department where injury occurred, type of sharp device caused injury, vaccination status of injured HCWs, and other detailed information regarding NSIs.

The hospital included 2165 Healthcare employees. The study includes physicians (consultants, specialists, and residents), nurses, other healthcare workers, and housekeepers. All healthcare workers exposed to sharp injuries must report their incidents to the infectious control office in the hospital according to the Ministry of Health policy. Staff within the infectious control office recorded detailed information about each NSIs in EPINet. Consent for data collection was provided from Dammam Medical Complex hospital director. The identities of participants were removed from the analysis data set to ensure privacy and dignity of HCWs.

The case definition of sharp devices in this study included disposable syringes, hypodermic needle, phlebotomy needles, other types of needles, scalpels, scissors, razor blade, and other sharp devices.

Data from EPINet software were coded and entered Excel and then imported to SPSS. Statistical analyses were done via SPSS software, V16. Chi-squared tests or fisher exact test (if the cells less than 5) were used in bivariate data analyses. *P*-value equal or less than 0.05 was considered statistically significant. The attack rate (incidence proportion) of NSIs was calculated by dividing the number of new cases of NSIs during the period of study by the population of HCWs at baseline.

## Results

There were 181 reported cases of NSIs from April 2016 to May 2018. NSIs were reported by 8.4% of the healthcare workers with available employment data in Dammam Medical Complex. Among the reported NSIs incidents, most injuries occurred among nurses (52.5%), followed by physicians (24.9%), other healthcare workers (which include non-lab technologist, clinical laboratory workers, respiratory therapist, and others) (16.6%), and housekeepers (6.1%).

Attack rates according to job title were calculated for occupations (Table 1). Nurses comprised the highest percentage of healthcare workers. The Dammam Medical Complex included 1235 nurses, 522 physicians, 340 housekeepers, and 68 other healthcare workers. The attack rate of NSIs in nurses during period of study was 7.7% (95 incidents among 1235 nurses), while the attack rate among physicians 8.6% (45 incidents among 522 physicians). The attack rate of NSIs among housekeepers is 3.2% (11 incidents among 340 housekeepers), and the attack rate in other healthcare workers was 44.1% (30 incidents in 68 workers). While nurses accounted for the most NSIs (52.5%), their individual risk was low (7.7%). The attack rates of NSIs among physicians and nurses were similar, but slightly higher among physicians (8.6%). The rate of NSIs among other HCWs was high (44.1%), and the attack rate in housekeepers was the lowest (3.2%). Also place of injury (the most common place of injury was ward, 32%) and associated activity with the injury (the most common activity associated with injury was during use, 32.6%) were shown in Table 1.

**Table 1:** The attack rate and frequency of NSIs

Characteristics	Number of cases	
**Job category**		**Attack rate**
Nurses	95	7.7
Physicians	45	8.6
Housekeepers	11	3.2
Other HCWs	30	44.1
**Place of injury**		**Percentage (%)**
Wards	58	32.1
ER	46	25.5
OR	18	9.9
Clinics	14	7.7
Dialysis and ICU	14	7.7
Other	31	17.1
**Activity associated with injury**		**Percentage (%)**
During use	50	32.6
While recapping	29	16.0
Device left	10	5.5
While disposal	12	6.6
After disposal	21	11.7
Other	59	27.6

Among all job categories, Needlestick injuries occurred mainly during the morning shift (68%). The night shift was the second most common shift for NSIs (23.2%), while the afternoon shift occurrence of NSIs was only 8.8%. Although the distribution of shifts by job type was not statistically significant, the percentage of NSIs occurrences during the night shift were highest among nurses, compared to other healthcare workers (Table 2).

**Table 2:** Association between job category and the time of the injury

Shift /Job	Physicians (%)	Nurses (%)	Housekeepers (%)	Other HCWs (%)	Total (%)
Morning shift	32 (71.1)	61 (64.2)	7 (63.6)	23 (76.7)	123 (68)
Afternoon shift	6 (13.3)	6 (6.3)	2 (18.2)	2 (6.7)	16 (8.8)
Night shift	7 (15.6)	28 (29.5)	2 (18.2)	5 (16.7)	42 (23.2)
Total	45 (100)	95 (100)	11 (100)	30 (100)	181 (100)

This table show the healthcare workers distributions of needlestick injuries according to 181 reported cases. Pearson chi-square 7.002, p-value 0.321

The work location of NSIs is summarized in Table 3. Most NSIs occurred at the wards (32%) or emergency rooms (25.4%). The work location of NSIs differed significantly by occupation (*p* <  0.001). Among the physicians, the injuries occurred most frequently in the emergency department (33.3%) and operating room (24.4%). Nurses most frequently experience sharp device injuries in wards (44.2%) and the emergency room (28.4%).

**Table 3:** Factors associated with NSIs

	Physicians (%)	Nurses (%)	Housekeepers (%)	Other HCWs^**1**^ (%)	Total (%)	*p- value
**Department of Injury**						
Wards	9 (20.0)	42 (44.2)	3 (27.3)	4 (13.3)	58 (32.0)	< 0.001
ER ^2^	15 (33.3)	27 (28.4)	1 (9.1)	3 (10.0)	46 (25.4)	
OR ^3^	11 (24.4)	6 (6.3)	1 (9.1)	0 (0.0)	18 (9.9)	
Clinics	6 (13.3)	3 (3.2)	1 (9.1)	4 (13.3)	14 (7.7)	
Dialysis and ICU ^4^	2 (4.4)	10 (10.5)	2 (18.2)	0 (0.0)	14 (7.7)	
Other	2 (4.4)	7 (7.4)	3 (27.3)	19 (63.3)	31 (17.1)	
Total	45 (100)	95 (100)	11 (100)	30 (100)	181 (100)	
**Action Associated with Injury**						
During use	29 (64.4)	15 (15.8)	0 (0.0)	6 (20.0)	50 (27.6)	< 0.001
While recapping	5 (11.1)	18 (18.9)	0 (0.0)	6 (20.0)	29 (16.0)	
Device left ^5^	1 (2.2)	7 (7.4)	1 (9.1)	1 (3.3)	10 (5.5)	
While disposing	0 (0.0)	9 (9.5)	1 (9.1)	2 (6.7)	12 (6.6)	
After disposal	1 (2.2)	17 (17.9)	1 (9.1)	2 (6.7)	21 (11.6)	
Other	9 (20.0)	29 (30.5)	8 (72.7)	13 (43.3)	59 (32.6)	
Total	45 (100)	95 (100)	11 (100)	30 (100)	181 (100)	
**Device Caused Injury**						
Disposable syringe	15 (33.3)	56 (58.9)	1 (9.1)	9 (30.0)	81 (44.8)	< 0.001
Needle on IV line	0 (0.0)	6 (6.3)	1 (9.1)	1 (3.3)	8 (4.4)	
Catheter needle	3 (6.7)	13 (13.7)	1 (9.1)	1 (3.3)	18 (9.9)	
Other needle	9 (20.0)	18 (18.9)	6 (54.5)	11 (36.7)	44 (24.3)	
Surgical	18 (40.0)	2 (2.1)	2 (18.2)	8 (26.7)	30 (16.6)	
Total	45 (100)	95 (100)	11 (100)	30 (100)	181 (100)	
**Gloves use**						
Single pair of gloves	39 (86.7)	70 (73.7)	9 (81.8)	19 (63.3)	137 (75.7)	0.132
Double pair of gloves	4 (8.9)	5 (5.3)	1 (9.1)	4 (13.3)	14 (7.7)	
No gloves	2 (4.4)	20 (21.1)	1 (9.1)	7 (23.3)	30 (16.6)	
Total	45 (100)	95 (100)	11 (100)	30 (100)	181 (100)	

This table show the different factors associated with needlestick injuries according to job category

^1^ Healthcare workers (HCWs), ^2^ Emergency room (ER), ^3^ Operation room (OR), ^4^ Intensive care unit (ICU), ^5^ Device left on the floor, table, or other inappropriate places

*p-value from chi-square or fisher exact test (if the cells less than 5)

As seen in Table 3, the actions associated with injuries are diverse. Among all health care workers, incidents during use of the sharp item (27.6%) and while recapping (16%) were common. However, a third of the incidents were classified as “other action,” which includes during steps of multi-step procedures, after use before disposal, in preparation for reuse of reusable instruments, withdrawing a needle from rubber or other resistance, restraining patient, disassembling device or equipment or before use of the item.

There were highly significant differences in associated actions according to occupation (*p* <  0.001) (see Table 3). Notably, a high proportion of NSIs did not fall into one of the pre-specified classes of actions and were therefore coded as “other”. Nurses more frequently experienced injuries because of “other” activities (30.5%), followed by while recapping the needles (18.9%). However, physicians more frequently experienced injuries during the use of the sharp items (64.4%), followed by “other” activities (20%).

Most of the NSIs occurrences are due to disposable syringes, accounting for 44.8% of the NSIs (See Table 3). However, there were significant differences according to occupation (*p* <  0.001). While disposable syringes are the primary source of NSIs among nurses (58.9%), surgical devices (e.g., scalpel and suture needles) play important roles as the most common devices that caused injury among the physicians (40%). Among housekeepers and the other health care workers, most NSIs occurred due to needles.

Table 3 summarizes glove use at the time of the NSIs. 16.6% of the HCWs were not wearing any gloves as personal protective equipment during their NSIs, while 75.7% of the HCWs were wearing a single pair of gloves. Only a small proportion of the HCWs were wearing double pair of gloves as protective method (7.7%). Although the association between gloves use and job category was not statistically significant, healthcare workers much more frequently wear a single pair of gloves during handling sharp devices. Physicians and nurses differed in use of double gloving. More physicians (8.9%) were wearing double pair of gloves compared to nurses (5.3%). Also, a higher percentage of nurses did not wear any gloves as protective equipment (21.1%) compared to physicians who did not wear gloves during their NSIs (4.4%).

Expatriates with NSIs are less likely than Saudi employees to have had a complete hepatitis B vaccination series before injury (Table 4). About one quarter of non-Saudi workers have not completed the three doses of hepatitis B vaccine (25.6%), compared to only 12.7% among Saudi workers (*p* = 0.047).

**Table 4:** Association between the nationalities of workers with NSIs and vaccination against Hepatitis B

Completed Hep B vaccination / Nationality	Saudi (%)	Non-Saudi (%)	Total (%)
Completed the vaccination	124 (87.3)	29 (74.4)	153 (84.5)
Uncompleted vaccination	18 (12.7)	10 (25.6)	28 (15.5)
Total	142 (100)	39 (100)	181 (100)

This table shows the number and percentage of persons with completed 3 doses of hepatitis B vaccination prior to the incident according to nationality. Pearson chi-square = 3.933, *p* = 0.047. Hep B = Hepatitis B

## Discussion

Our retrospective study using an administrative database (EPINet software) found that sharp device injuries were frequent. These injuries affected a significant proportion of healthcare workers (8.4%) and affected multiple healthcare professions, especially nurses and physicians. This incidence in this study (8.4%) was very low when compared to a study (19.5%) [10] or to a global pooled prevalence of NSIs among HCWs (44.5%) [11]. In this study, the category “other HCWs” had approximately 6 times higher risk than nurses, and physicians had 1.11% higher risk than nurses. These differences are most likely due to under-reporting, or the present study is based on voluntary reporting only. In this study, the hospital has occupational health and safety program to address this problem. However, there are no penalty for those who have work accidents and there is no cost for prophylaxis after a NSI.

We noted different patterns for physicians and for nurses among the participants. We also found certain employee groups had inadequate pre-exposure immunizations. In this study, nurses account for the greatest proportion of NSIs. This may be related to the nature of their job. Nurses have frequent close contact with the patients, they perform most of the procedure with the sharp items, including phlebotomy, IV needle insertion, and performing injections. The proportion of NSIs in nurses (52.5%) is similar to the proportion of nurses in the at-risk workforce (1235/2165, or 57%). The high percentage of NSIs among nurses can be attributed to their large number rather than an increased risk per person. The physicians are the second job category affected by NSIs. Studies showed that nurses are most affected job category for Needlestick injuries with prevalence ranging from 36 to 72.7% [2, 12–17].

Among all job categories, NSIs occurred most frequently during the morning shift. That corresponds to the highest level of activities of medical practice, the largest number of patients, and the largest number of healthcare workers in the morning shift. The night shift followed the morning shift in the occurrence of NSIs, possibly because the workers are contained and sleepy. This agrees with a study [18], that the number of injuries increased during the morning shift.

This study found that sharp device injuries occurred most frequently in the wards, followed by emergency rooms (ER), then operating rooms (OR). This result is similar to other studies [19, 20], that show the wards are the most common place of NSIs occurrence (65.6%). Among nurses, most NSIs occur in the wards followed by ER, while among physicians most of the NSIs occur in the ER, followed by OR. That can be explained by the nature of the work and the medical activities for each job category. In patient wards, nurses perform with sharp objects, including IV access procedures and different types of injections. Physicians do most of their procedures with sharp devices including scalpel and suture needle in operating rooms.

The frequency of NSIs (affecting 8.4% of workers) underscores the importance of building programs to prevent NSIs. Such approaches include training about standard precautions, assuring use of personal protective equipment such as wearing gloves, recapping prohibition, special containers for sharp objects, HCWs immunization, and post-exposure prophylaxis. Our data suggest carefully considering whether a single sharp device injury prevention program will be effective for all workers or, alternatively, if program elements should be targeted according to risk group. Advantages of specific targeting include improving cost-effectiveness for intensive intervention programs as well as strongly encouraging each person to use the best methods for his/her specific work.

Very few programs employ targeted approaches. For example, programs [21] for training of HCWs, and the adoption of needle-safety devices, have an impact on preventing NSIs. The US Occupational Safety and Health Administration (OSHA) suggests that general work practice control and engineering control for all HCWs are the primary means that should be used to reduce risks of NSIs [1].

Programs should emphasize specific causes of NSIs. NSIs while recapping was common (16% overall). Guidelines to reduce such injuries may include prohibition of recapping, use of the mechanical recapping devices, or a one hand technique. A recent review [22] of the effectiveness of safety engineered injection devices found there was moderate quality evidence that using this safety engineered injection devices reduces the incidence of needlestick injuries among healthcare workers.

In contrast to nurse-oriented programs, programs for physician should emphasize surgical devices as well as syringes. Another study [14] showed that nurses are most commonly stuck with hollow-bore needle (78%), while physicians are most commonly stuck by devices such as scalpels and suture needles (67.6%).

In addition, the use of blunt needles reduces the risk of contracting infectious diseases for surgeons by reducing the number of needlestick injuries. A Cochrane Database Systemic Review [23] of 10 randomized controlled trials compared surgeons who used blunt suture needles and surgeons who use sharp needles. The review concluded that there was high quality evidence that the use of blunt needles will reduce the hazard of exposure to blood and other body fluids.

Our study suggested that expatriate healthcare workers with NSIs are less likely than Saudi employees to have had a complete hepatitis B vaccination series before injury. About a quarter of injured non-Saudi were not vaccinated or had not completed the three doses of hepatitis B vaccine before the NSIs. Expatriates’ housekeepers have rapid turnover and face education and language barriers. Verifying that all employees have received three doses of hepatitis B vaccine prior to starting work is important particularly for the expatriate workers despite their rapid turnover. Hepatitis B vaccination protects workers from risks of the life-threatening consequences of hepatitis B infection [24]. In Saudi Arabia, a program for hepatitis screening for expatriates was established years ago [25]. A study done in Saudi Arabia [26] to assess vaccination status in blue collar workers showed 40% of participants had not been vaccinated against hepatitis B. It also showed a significant association between high level of knowledge and vaccination status.

Compliance with standard precautions such as wearing gloves or other personal protective equipment must also strengthened employee training. In this study, most of the HCWs wore only a single pair of gloves during their injuries, although wearing double pairs of gloves decreases chances of percutaneous exposure incidents [27]. In our study, 16.6% of the HCWs were not using gloves at the time of their sharp injuries.

A Cochrane Evidence Review considered 34 RCTs [27] about the effectiveness of additional gloves to decrease incidence of percutaneous exposures among HCWs. There was a moderate quality evidence that double pairs of gloves will decrease the risk of gloves puncture and decrease the risks of skin blood stains when compared to single pair of gloves.

Prevention requires effective surveillance systems. Most depend upon administrative databases rather than self-reported injuries from workers, suggesting incomplete data recording. However, surveillance techniques are not consistently applied. Monitoring is necessary to evaluate the effectiveness of programs and to compare alternative approaches (e.g., discipline specific versus generic programs). The use of non-disposable versus disposable and safe syringes showed a significant reduction in avoidable needlestick injuries. In addition, there was a significant reduction in cost for management of needlestick injuries, which include workers psychological problems after injury [28–30].

Prevention program should be established in the hospital to reduce incidence and risk of NSIs among HCWs, and to reduce risk of viral infections transmission by these injuries. There is increasing need to implement the use of sharp device with safety engineering controls that help to reduce the risk of NSIs. All HCWs should be trained to use these devices with safety features and should be properly trained how safely handle sharp equipment. In addition, needles recapping should be avoided, and disposing used sharp devices in sharp disposal containers should be appropriate to prevent injury.

This study has limitations due to underreporting of NSIs incidents since the data were obtained from an administrative database rather than the workers themselves. Reasons for not reporting include lack of knowledge of the benefit of post-exposure prophylaxis, fear that the reporting will affect the career, belief that there is very low risk of transmission of infection, and time-consuming reporting methods. It is necessary that all HCWs appreciate the importance of reporting their NSIs incidents and that disincentives to reporting be removed.

Also, the EPINet (Exposure Prevention Information Network) software has a limited information. The characteristics of participants, work environment, work organization, schedules, work hours, stress, previous training, or other occupational risks were not available in the software as per hospital policy. These information were classified as confidential and can only be accessed by the hospital management through other system such as personal records. This study was susceptible to survivor bias because it did not consider those who had retired or resigned. Furthermore, the participants were recruited from one hospital and that the generalizability of the findings may be limited because of the small sample size.

## Conclusions

This study of a large Saudi Arabia Medical Center describes characteristics and factors associated with NSIs. Our data indicated that sharp device injuries were frequent among the participants were nurses account for proportion of NSIs. These injuries occurred most frequently during the morning shift and most common in the wards as nurses perform with sharp objects. Expatriate healthcare workers with NSIs are less likely than Saudi employees to have had a complete hepatitis B vaccination series before injury. The findings of this study may guide the development of programs targeted to specific risk categories in order to more effectively prevent these injuries. The hospital must provide and enforce the use of engineering controls such as needles systems and must arrange workshops for HCWs to train them on preventive measures and to discourage underreporting.

## Data Availability

The datasets generated and/or analyzed during the current study are not publicly available due to the studied hospital policy but are available from the corresponding author on reasonable request.
